# Assessment of the Neutralizing Antibody Response of BNT162b2 and mRNA-1273 SARS-CoV-2 Vaccines in Naïve and Previously Infected Individuals: A Comparative Study

**DOI:** 10.3390/vaccines10020191

**Published:** 2022-01-25

**Authors:** Farah M. Shurrab, Duaa W. Al-Sadeq, Haissam Abou-Saleh, Nader Al-Dewik, Amira E. Elsharafi, Fatima M. Hamaydeh, Bushra Y. Abo Halawa, Tala M. Jamaleddin, Huda M. Abdul Hameed, Parveen B. Nizamuddin, Fathima Humaira Amanullah, Hanin I. Daas, Laith J. Abu-Raddad, Gheyath K. Nasrallah

**Affiliations:** 1 Biomedical Research Center, Qatar University, Doha P.O. Box 2713, Qatar; farah.shurrab@qu.edu.qa (F.M.S.); da1206066@student.qu.edu.qa (D.W.A.-S.); hasaleh@qu.edu.qa (H.A.-S.); parveen.n@qu.edu.qa (P.B.N.); fa1517855@student.qu.edu.qa (F.H.A.); 2 College of Medicine, QU Health, Qatar University, Doha P.O. Box 2713, Qatar; 3 Biological Science Program, Department of Biological and Environmental Sciences, College of Arts and Science, Qatar University, Doha P.O. Box 2713, Qatar; ba1702821@student.qu.edu.qa; 4 Women’s Wellness and Research Center (WWRC), Clinical and Metabolic Genetics Section, Pediatrics Department, Hamad General Hospital (HGH), Interim Translational Research Institute (iTRI), Hamad Medical Corporation (HMC), College of Health and Life Science (CHLS), Hamad Bin Khalifa University (HBKU), Doha P.O. Box 3050, Qatar; dewik2000@yahoo.com; 5 Department of Biomedical Science, College of Health Sciences, QU Health, Qatar University, Doha P.O. Box 2713, Qatar; ae1705431@student.qu.edu.qa (A.E.E.); fa1802833@student.qu.edu.qa (F.M.H.); tj1800900@student.qu.edu.qa (T.M.J.); ha1803800@student.qu.edu.qa (H.M.A.H.); 6 College of Dental Medicine, QU Health, Qatar University, Doha P.O. Box 2713, Qatar; hdaas@qu.edu.qa; 7 Infectious Disease Epidemiology Group, Weill Cornell Medicine-Qatar, Cornell University, Qatar Foundation—Education City, Doha P.O. Box 24144, Qatar; lja2002@qatar-med.cornell.edu; 8 World Health Organization Collaborating Centre for Disease Epidemiology Analytics on HIV/AIDS, Sexually Transmitted Infections, and Viral Hepatitis, Weill Cornell Medicine–Qatar, Cornell University, Qatar Foundation—Education City, Doha P.O. Box 24144, Qatar; 9 Department of Healthcare Policy and Research, Weill Cornell Medicine, Cornell University, New York, NY 14850, USA

**Keywords:** neutralizing antibodies, Pfizer-BNT162b2, Moderna-mRNA-1273, SARS-CoV-2, vaccine

## Abstract

The currently authorized mRNA COVID-19 vaccines, Pfizer-BNT162b2 and Moderna-mRNA-1273, offer great promise for reducing the spread of the COVID-19 by generating protective immunity against SARS-CoV-2. Recently, it was shown that the magnitude of the neutralizing antibody (NAbs) response correlates with the degree of protection. However, the difference between the immune response in naïve mRNA-vaccinated and previously infected (PI) individuals is not well studied. We investigated the level of NAbs in naïve and PI individuals after 1 to 26 (median = 6) weeks of the second dose of BNT162b2 or mRNA-1273 vaccination. The naïve mRNA-1273 vaccinated group (*n* = 68) generated significantly higher (~2-fold, *p* ≤ 0.001) NAbs than the naïve BNT162b2 (*n* = 358) group. The P -vaccinated group (*n* = 42) generated significantly higher (~3-fold; *p* ≤ 0.001) NAbs levels than the naïve-BNT162b2 (*n* = 426). Additionally, the older age groups produced a significantly higher levels of antibodies than the young age group (<30) (*p* = 0.0007). Our results showed that mRNA-1273 generated a higher NAbs response than the BNT162b2 vaccine, and the PI group generated the highest level of NAbs response regardless of the type of vaccine.

## 1. Introduction

Coronavirus disease 2019 (COVID-19) has emerged as a global pandemic, causing significant widespread morbidity and mortality. The severe acute respiratory syndrome coronavirus-2 (SARS-CoV-2) has infected over 100 million individuals, with more than five million deaths [1]. The current COVID-19 epidemiological status in Qatar at the time of this study (3 to 17 January 2022) is as follows: 299,242 confirmed cases, 626 deaths, and 5,447,278 vaccine doses have been administered (WHO) [2]. The total population of Qatar is ~2.6 million. As of today, 84.5% of the Qatari population has received at least two doses of the vaccines (either BNT162b2 or mRNA-1273) and around 10% of the population has received the third dose.

The COVID-19 pandemic has launched an intensive effort worldwide to develop efficient vaccines. As a result, different technologies have been used to develop effective vaccines, including mRNA-based vaccines such as BNT162b2 (Pfizer-BioNTech) and mRNA-1273 (Moderna), viral vector vaccines such as ChAdOx1 (Oxford/AstraZeneca), and inactivated vaccines such as Sinovac and Sinopharm [3]. BNT162b2 and mRNA-1273, obtained Emergency Use Listing (EUL) from WHO and have demonstrated a high degree of protection against the COVID-19 illness [4,5]. These vaccines induce antibodies to the SARS-CoV-2 spike protein (S-protein), including NAbs against the receptor-binding domain (RBD) of the S-protein (S-RBD) [6]. To have a general idea about the immune response to the aforementioned vaccines, we recently analyzed the binding antibody (total antibodies, IgG, and IgA) response to anti-SARS-CoV-2 antibodies to S-RBD [7]. Because it is evident now that the level of vaccine-induced NAbs response correlates better with the degree of protection than the binding antibodies [8,9,10], here, we further investigated the NAbs response generated after vaccination with the BNT162b2 or the mRNA-1273 vaccines in naïve and previously infected (PI) groups.

## 2. Material and Methods

### 2.1. Ethical Approval and Sample Collection

Randomized participants who received two BNT162b2 or mRNA-1273 vaccine doses were eligible for inclusion. A total of 468 blood samples were collected from staff and students at Qatar University, the national university in Qatar, with different age groups and nationalities. Peripheral blood was collected 1–26 weeks (BNT162b2 median = 9, mRNA-1273 median = 6, PI median = 9) following the administration of the second dose of the vaccine. Participants were naïve or PI with SARS-CoV-2. The median duration between previous infection and vaccination with the second dose was 22 weeks. In addition, demographic information, including the previous history of infection, was collected through a self-administered questionnaire. The study was reviewed and approved by the Institutional Review Board at Qatar University (QU-IRB 1537-FBA/21). Plasma was separated from venous whole blood collection and stored at −80 °C until the immunoassays.

### 2.2. Serology Testing

NAbs testing was carried out using the CL-900i^®^ automated analyzer from Mindray Bio-Medical Electronics. The performance CL-9000i^®^ was previously validated [11,12,13]. The CL-900i^®^ NAbs assay is a chemiluminescence immunoassay, where NAbs in the sample compete with ACE2-ALP conjugate for the binding sites of SARS-COV-2 S-RBD immobilized in the paramagnetic microparticles. According to the WHO standards, the conversion factor of this assay is 1 AU = 3.31 IU/mL, and the reference range is from 10 to 400 AU/mL (Ref.: SARS-CoV-2 Neutralizing Antibody 121, Mindray, China). All samples with readings higher than the reference range were diluted with phosphate-buffered saline (PBS). In addition, all samples were tested using the Architect automated chemiluminescent assay (Abbott Laboratories, USA) to detect anti-N SARS-CoV-2 IgG antibodies; the test was carried out according to the manufacturer’s instructions [13]. Therefore, PI status was classified as anti-N IgG positive results and/or having confirmed positive RT-PCR results collected from the questionnaire.

### 2.3. Statistical Analysis

Data were analyzed using GraphPad Prism 9.2.0. (San Diego, CA, USA). D’Agostino–Pearson’s test for normal distribution was performed, followed by a one-way ANOVA test (Kruskal–Wallis with Dunn’s multiple comparisons). The results in the graphs were plotted as mean values with the standard deviation (SD), and *p* values < 0.05 were considered statistically significant. In all graphs, significance is marked as * if *p* ≤ 0.05, ** if *p* ≤ 0.01, and *** if *p* ≤ 0.001.

## 3. Results

### 3.1. Participant Characteristic

The participants’ characteristics are described in Table 1. In total, 468 naïve mRNA-vaccinated and PI vaccinated volunteers participated in this study. Of these, 358 were naïve BNT162b2-vaccinated, 68 were naïve mRNA-1273-vaccinated, and 42 were PI (34 with BNT162b2 and eight with mRNA-1273) vaccinated participants.

### 3.2. Neutralizing Antibody Response Assessment 

**Figure 1:**
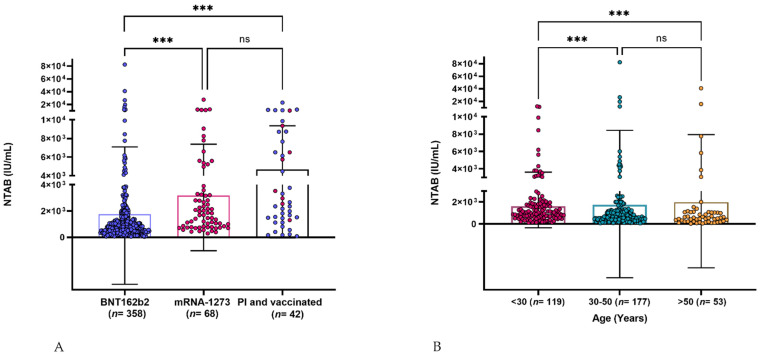
(**A**) NAb levels (IU/mL) in mRNA vaccinated participants 1–26 weeks after receiving two doses, and participants with prior infection and two doses of the vaccine. (**B**) NAb levels (IU/mL) in BNT162b2-vaccinated participants according to age groups. The tests were performed using the automated analyzer Mindray Cl-900i. Asterisks on graphs denote * *p* ≤ 0.05, ** *p* ≤ 0.01, and *** *p* ≤ 0.001; ns *p* > 0.05.

All naïve mRNA-1273- and PI vaccinated groups had a 100% positive NAbs response, whereas the response in the naïve BNT162b2-vaccinated group was 99.7%. The mean levels were 1.8 × 10^3^ IU/mL (95% CI: 1.2–2.3 × 10^3^) for the naïve BNT162b2 group, 3.2 × 10^3^ IU/mL (95% CI: 2.2–4.2 × 10^3^) for the naïve mRNA-1273 group, and 4.7 × 10^3^ IU/mL (95% CI: 3.1–6.1 × 10^3^) for the PI group. The naïve mRNA-1273 vaccinated group generated a significantly higher level of NAbs than the naïve BNT162b2 group (~2-fold, *p* ≤ 0.001). The PI vaccinated group generated significantly higher NAbs levels than the naïve BNT162b2 (~3-fold; *p* ≤ 0.001). However, no significant difference was observed between the PI vaccinated group and the naïve mRNA-1273 group (*p* = 0.7718) (Figure 1A).

### 3.3. Age Effect on Neutralizing Antibody Response

Samples from the naïve BNT16b2-vaccinated participants (*n* = 349) were categorized into different age groups: <30, 30–50, and >50 years old. The median age in each group was 22 (interquartile range (IQR): 21–26 years), 40 (IQR: 35–45 years), and 56 (IQR: 53–61 years) years, respectively. Each group’s mean NAbs level was 1.6 × 10^3^ IU/mL (95% CI: 1.3–1.9 × 10^3^), 1.8 × 10^3^ IU/mL (95% CI: 7.7 × 10^2^–2.8×10^3^), and 2.0 × 10^3^ IU/mL (95% CI: 3.3 × 10^2^–3.6 × 10^3^), respectively. The older age groups produced a significantly higher levels of antibodies than the young age group (<30) (*p* = 0.0007) (Figure 1B).

## 4. Discussion

Both PI and naïve vaccinated individuals showed robust immune responses to vaccination. However, this response appeared to be significantly higher in PI individuals, especially when compared with the naïve BNT162b2 group (Figure 1A). Our data are in concordance with previously published reports [14,15,16]. The acquired immunity from infection is boosted by vaccination. Memory B cells are likely responsible for providing a robust, immediate recall of high NAbs in PI individuals [17]. However, we noticed that some naïve vaccinated individuals could have a high level of NAbs response against SARS-CoV-2 (Figure 1A, BNT162b2-vaccinated group). The highest level of NAbs reached 8.2 × 10^4^ IU/mL, which a naïve BNT162b2-vaccinated individual generated. This robust immunity could be due to a particular genetic makeup or prior undetected asymptomatic infection. A recent computational study indicated that a set of major histocompatibility complex (MHC) genes, which are essential for the adaptive immune response, help to regulate the strength and durability of NAbs to SARS-CoV-2 [18].

Different mRNA vaccine products also resulted in different NAb responses. For instance, the level of NAbs evoked in the naïve mRNA-1273 group was significantly higher compared with the BNT162b2 group. These results are consistent with Tyner et al., who demonstrated that the mRNA-1273 vaccine generated higher NAbs titers when measured using a pseudovirus neutralization assay [19]. This can be explained by the fact that different vaccine formulations, dose contents, and intervals between doses may contribute to the varying levels of NAbs production between mRNA-1273 and BNT162b [20]. Although there was a twofold difference in NAbs levels between mRNA-1273 and BNT162b post-vaccination, both mRNA vaccines showed high comparable efficacy against SARS-CoV-2 infection [4,5]. The wide interval (1–26 weeks) of sample collection between participants could have influence our results. However, we believe that this should have a minimal effect, as previous studies have shown that antibody levels start to decline 13–26 weeks after receiving the second dose [15,21,22].

The response of antibody levels to vaccination declined with age [23]. However, our serologic results showed an increasing trend with age. A previous study showed a minimal effect of age on antibody responses after vaccination [24]. This could be explained by the fact that BNT162b2 induces a high immune response, especially after two doses. Therefore, it elicited high NAbs levels regardless of age. According to Jalkanen et al., the level of antibodies was significantly lower in elderly individuals (>50 years) compared with younger ones after receiving the first dose of BNT162b2. Nevertheless, this difference diminished upon receiving the second dose [24,25]. The observed increasing trend in our results can be explained by the higher frequency of SARS-CoV-2 spike-specific memory B cells’ response in the elderly group after receiving the second dose [26].

This study has some limitations. The immune response after the first dose needs further investigation, especially in the PI group. In addition, the kinetics and longevity of these NAbs should be studied in the future. Lastly, although NAbs were proven to correlate with protection [8], the minimum required levels of NAbs to provide protection are not yet determined. Additionally, further investigation of the memory B and T cells’ responses after vaccination is needed. That is, it has been reported that vaccinated individuals may have continued protection against the diseases as a result of durable memory B and T cells [27].

## 5. Conclusions

In conclusion, our results showed that PI individuals generated higher NAbs levels compared with naïve vaccinated participants after receiving two doses of the mRNA vaccine. However, although both vaccines showed an excellent NAbs response against SARS-CoV-2, it is too early to make any firm conclusions based on these findings with implications for public health vaccine policy.

## Figures and Tables

**Table 1:** Demographic characteristics of the study sample (*n* = 468).

	Characteristic	BNT162b2 N (%)		mRNA-1273 N (%)	
Gender		Naïve	PI	Naïve	PI
	Male	170 (47.5)	10 (29.4)	27 (39.7)	3 (37.5)
	Female	175 (48.9)	15 (44.1)	41 (60.3)	5 (62.5)
	Unknown	13 (3.6)	9 (26.4)	-	-
	Total	358	34	68	8
Age (years)	>30	119 (33.2)	15 (44.1)	41 (60.3)	3 (37.5)
	30–50	177 (49.4)	17 (50)	25 (36.8)	5 (62.5)
	>50	53 (14.8)	2 (5.8)	2 (2.9)	-
	Unknown	9 (2.5)	-	-	-
	Total	358	34	68	8

## Data Availability

Derived data supporting the findings of this study are available from the corresponding author upon request.
